# Functional Identification and Characterization of Leucokinin and Its Receptor in the Fall Webworm, *Hyphantria cunea*

**DOI:** 10.3389/fphys.2021.741362

**Published:** 2021-10-07

**Authors:** Lili Sun, Heting Ma, Yuan Gao, Zhiying Wang, Chuanwang Cao

**Affiliations:** Key Laboratory of Sustainable Forest Ecosystem Management-Ministry of Education, College of Forestry, Northeast Forestry University, Harbin, China

**Keywords:** G-protein-coupled receptor, *Hyphantria cunea*, leucokinin, RNA interference, gene function

## Abstract

Neuropeptides function as central neuromodulators and circulating hormones that modulate insect behavior and physiology. Leucokinin (LK) is an intercellular signaling molecule that mediates many physiological and behavioral processes. However, the functions of LK associated with environmental stress and feeding behavior in the fall webworm, *Hyphantria cunea*, is little known. Our primary objective is to understand the function of LK and LK receptor (LKR) neuroendocrine system in *H. cunea*. In the present study, the results showed that LK/LKR are expressed at different developmental stages and in various tissues of *H. cunea*. A candidate receptor–ligand pairing for LK was identified in the larval transcriptome of *H. cunea*. In a heterologous expression system, the calcium assay was used to demonstrate that LKR is activated by HcLKs in a dose-dependent manner, with 50% effective concentration (EC_50_) values of 8.44–90.44nM. Knockdown of *HcLK* and *HcLKR* by microinjecting target-specific dsRNA leads to several effects in *H. cunea*, including feeding promotion, increase in resistance to desiccation and starvation stress, and regulation of water homeostasis. The transcript levels of *HILP2* (except in the *LK* knockdown group), *HILP5*, and *HILP8* increased, whereas those of *HILP3*, *HILP4*, and *HILP6* decreased; *HILP1*, *HILP2* (in the LK knockdown group), and *HILP7* gene expression was not influenced after *LK* and *LKR* knockdown. Variations in mRNA expression levels in insulin-like peptide genes in the knockdown larvae suggest an essential role of these genes in survival in *H. cunea*. To our knowledge, the present study is the first comprehensive study of LK and LKR – from gene to behavior – in *H. cunea*.

## Introduction

As the central neuromodulators and circulating hormones, neuropeptides orchestrate insect behavior and physiology. The complex hormonal and neuronal regulatory mechanisms maintain the metabolic homeostasis, which balance the food intake, energy expenditure, and nutrient storage in insects (Murphy and Bloom, 2006; Baker and Thummel, 2007; Leopold and Perrimon, 2007; Woods and D’Alessio, 2008; Teleman, 2010; Dalamaga et al., 2013; de Araujo et al., 2013; Vogt and Bruning, 2013). Mechanisms of feeding and metabolism have been explored in depth in *Drosophila melanogaster* (Baker and Thummel, 2007; Itskov and Ribeiro, 2013; Owusu-Ansah and Perrimon, 2014; Padmanabha and Baker, 2014), and it is known that food ingestion and metabolic homeostasis are mediated by several peptide hormones (Wu et al., 2003, 2005; Melcher and Pankratz, 2005; Géminard et al., 2006; Bharucha et al., 2008; Al-Anzi et al., 2010; Cognigni et al., 2011; Hergarden et al., 2012; Söderberg et al., 2012; Itskov and Ribeiro, 2013). Insect food ingestion is associated with a balance of water and ions (Coast et al., 2002; Dow and Davies, 2006; Dow, 2009). Thus, it is likely that insect diuretic hormones collaborate with the hormones released after food intake to regulate satiety, metabolism, and energy reallocation.

Kinins (leucokinins) in insects have a highly conserved C-terminal pentapeptide sequence – Phe-Xaa-Xbb-Trp-Gly-NH_2_, where Xaa represents Tyr, His, Ser, or Asn; Xbb may be Ala but is generally Ser or Pro (Holman et al., 1990, 1999). Insect leucokinins (LKs) are multifunctional peptides acting as neurohormones and neurotransmitters, which regulate diuresis, sleep, metabolism, response to ionic stress, food intake, and taste responsiveness (Terhzaz et al., 1999; Radford et al., 2002; Al-Anzi et al., 2010; Cognigni et al., 2011; López-Arias et al., 2011; Kwon et al., 2016; Zandawala et al., 2018a,b; Yurgel et al., 2019). In *D. melanogaster*, LK acts *in vitro* on stellate cells of the renal tubules to trigger fluid secretion, which is produced by a small set of neurons and neurosecretory cells in the central nervous system (CNS; de Haro et al., 2010). Leucokinins aid fluid excretion by increasing the secretion of primary urine by the Malpighian tubules and contracting the hindgut. Together with insulin signaling, the LK neuropeptide regulates stress tolerance and metabolism in *D. melanogaster* (Zandawala et al., 2018a).

The fall webworm *Hyphantria cunea* Drury (Lepidoptera: Noctuidae), a worldwide forest pest that originated in North America, was first reported in China in 1979 (Rong et al., 2003; Zhang et al., 2008). To alleviate the damage caused by *H. cunea*, various control strategies have been developed, such as natural predation, microbial intervention, and insecticide usage (Beckage, 2008). Because neuropeptides are regulators of critical life processes in insects and are highly specific, they are the potential targets in the development of green insecticides. The present study aims to understand the neuroendocrine pathways regulating the key physiological processes in pest insects for screening the potential analogs. The leucokinin signaling system has been studied in several other insect species; however, localization and functional roles of leucokinin in *H. cunea* remain unknown.

In this study, we first investigated the function of the LK ligand and receptor signaling system in *H. cunea*. Subsequently, we determined the transcript levels of the *LK* and LK receptor (*LKR*) genes under starvation to examine whether this signaling system was affected by the feeding behavior of *H. cunea*. *LK* gene knockdown *via* RNAi was used to further examine the potential relationship between LK signaling and the feeding behavior of *H. cunea*. We demonstrate that LK signaling regulates starvation stress and feeding.

## Materials and Methods

### Insects

*Hyphantria cunea* eggs and artificial diets were obtained from the Research Institute of Forest Ecology, Environment and Protection, Chinese Academy of Forestry (Beijing, China). Eggs were incubated at 25°C until hatching, and larvae were fed on artificial diets in 250ml transparent plastic bottles, which were maintained at 25±1°C with a 16:8h light:dark photoperiod.

### Molecular Cloning and Plasmid Construction

Reverse transcription PCR was initially used to validate the sequences of *H. cunea LK* and *LKR* transcripts from the *H. cunea* genome database. The *LK* and *LKR* genes were cloned using the following thermal conditions: 94°C for 3min; followed by 35cycles of 94°C for 30s, 60°C for 30s, and 72°C for 1min; then a final extension at 72°C for 10min. The PCR product was sub-cloned into pMD18-T vector (TaKaRa, Japan) and then verified sequences. The primers used for the PCR cloning of *HcLK* and *Hyphantria cunea* leucokinin receptor (*HcLKR*) are presented in Table 1. The PCR products were directly cloned into the pcDNA-3.1-myc-His vector. The recombinant vectors were verified by sequencing.

**Table 1:** List of primers used in this study.

Primer name	Primer sequence (5′ to 3′)		Primer usage
	Forward	Reverse	
HILP1	ATGAAGCGAGACGCTGGAT	TCAGGTCTGAAATTCTTTGGT	RT-qPCR
HILP2	GAAGTTTCTAATTGTAGTTCTTTCACT	TAGTTCATCAACAGTGCAAGGT	
HILP3	ATGGTGAAGCGGGATTCAG	TTAGCAGTATGTGAGCAGTTCA	
HILP4	ATGAAGGTGGCTCTAGCT	AGAAGTTCTTCAACAGTGCAAG	
HILP5	CTTTGCTTTAATGGCCGGTTA	CACGCTGTCGGACAAATC	
HILP6	ATGCTAGCGGCTTTGTGTT	CGAAGAATGCTGTGATAAGCC	
HILP7	ATGAAGTTCTCATTGGTGTTAGTC	GCAGTATGTGAGCAATTCATCA	
HILP8	CATTGGTCTATGGTTACGTATCAG	AGTAAGTGAGCAGTTCATCG	
qLK	ATGTTGCACCAATGGCTCATCATC	CATCGTCGCGTTGGTAAAACTG	
qLKR	TATTCCTCCCGGCGATATATTGAAAG	ACAATTCACTGACTCTCTCATCG	
RPL13	GTTAGCTACACAGCTCCGTGG	GCAGCAGTTGGGGCTTTAGT	
EF-1α	ATGAAATCTCTGTGACCGGGG	GCGGTGGTATCGACAAACGT	
LKR-pcDNA3.1	ATCG*GGATCC*ATGGACACCAGTACAGCAAATACTAC	CCC*AAGCTT*CACTTTGTCATCGTACGATACGTC	Cell transfected
dsLK	TAATACGACTCACTATAGGGAACCCTCATCTAGACACAGA	TAATACGACTCACTATAGGTCCTCTCGCTCGTTTTGG	RNAi
dsLKR	TAATACGACTCACTATAGGGAAGATGAACTAGATCCAGCTAC	TAATACGACTCACTATAGGACTATCGTTTACGTATCTGCTTGG	
dsEGFP	TAATACGACTCACTATAGGGGAGAAGAACTTTTCACTGG	TAATACGACTCACTATAGGAGTTGAACGGATCCATCTTC	
qGADPH	TTCAGCTCTGGGATGACCTT	TGCCACTCAGAAGACTGTGG	RT-qPCR of cell

### Analysis of LK and LKR

The deduced amino acid sequences of LK and LKR orthologs were obtained from GenBank using BLAST searches (blastx and tblastx). Multiple alignment of the amino acid sequences was performed using the ClustalX2 program and BioEdit. A phylogenetic tree was constructed using the neighbor-joining (NJ) method in MEGA 5.0 with 1,000 bootstrap replicates (Tamura et al., 2011). Signal peptides were predicted using Signal P 4.1 Server (Mccarthy et al., 2004), and transmembrane domains were predicted using TMHMM server v2.02 (Sonnhammer et al., 1998). The presence of N-glycosylation sites in predicted protein sequences was assessed using NetNGlyc 1.0^1^, and the generation of sequence logos for the C-terminal motifs of LK proteins was created by Weblogo (Crooks et al., 2004).

### Cell Culture and Transfection

The human embryonic kidney 293 (HEK293) cell line was cultured in Dulbecco’s modified Eagle medium supplemented with 10% fetal bovine serum (FBS) and 4mM L-glutamine (Invitrogen) at 37°C in a humidified incubator containing 5% CO_2_. HEK293 cells were transfected with *LKR* cDNA plasmid constructs using Effectene transfection reagent (Qiagen) according to the manufacturer’s instructions. Two days after transfection, stably expressing cells were selected by the addition of 800mg/L G418.

### Intracellular Calcium Assay

To investigate the interaction between the LKR and LKs in *H. cunea*, the response of the LKR to chemically synthesized LKs was examined using the Ca^2+^ imaging assay. A fluorescent Ca^2+^-sensitive probe, Fura-4/AM (Beyotime, Shanghai, China), was used to detect the intracellular cytosolic calcium signals according to the manufacturers’ instructions. In brief, HEK293 cells stably expressing LKR were washed twice with phosphate-buffered saline and were suspended at 5×10^6^ cells/ml in Hanks’ balanced salt solution. The cells were then loaded with 2μl Fura-4/AM for 20min and washed twice with HBSS buffered medium. Then, cells were stimulated with 0.1 and 1μM HcLKs (HcLK-1, HcLK-2, and HcLK-3) chemically synthesized by Sangon Biotech Co., Ltd. (Shanghai, China). Each 96-well plate was transferred into a Multi-Mode Microplate Reader (Varioskan Flash Beckman XL-70 F; Thermo Fisher Scientific Inc. Waltham, MA) to monitor the Fluo-4 fluorescence. The excitation wavelength was 485nm, and fluorescence emission was detected at 520nm. Various concentrations of receptor ligands were added when Fluo-4 fluorescence had reached a stable value in each well. The changes in Fluo-4 fluorescence were recorded automatically. Dose–response curves for putative agonists were established in at least three independent experiments.

### RNA Interference

A 463-bp dsRNA representing the *H. cunea* LK-encoding gene sequence and a 505-bp dsRNA representing the *H. cunea* LKR-encoding gene sequence were synthesized using the MEGAscript T7 high-yield transcription kit (Ambion) according to the manufacturers’ protocol. The dsRNA was purified with phenol/chloroform followed by ethanol precipitation. The dsRNA of the enhanced green fluorescent protein gene (pEGFP-N1 plasmid as template, WP_031943942.1, 507-bp dsRNA) was employed as a control. A 2μg/μl dsRNA solution (1μl) was microinjected into the penultimate posterior abdominal section of individual seventh instar *H. cunea* larvae using an injection needle (MICROLITERTM #65 with 33-gauge needle, Hamilton Co., Reno, NV, United States) under ice anesthesia (Sun et al., 2016). Control *H. cunea* larvae were microinjected with the *EGFP* dsRNA. Microinjected *H. cunea* larvae were allowed to recover for 2h at room temperature and then reared on an artificial diet under a 16:8h light: dark photoperiod at 25±1°C. After 72 and 96h, *LK* and *LKR* mRNA levels in the dsRNA-treated seventh instar *H. cunea* larvae were measured by qRT-PCR technology.

### Bioassays

To measure water content, the larvae treated with *dsEGFP*, *dsLK*, and *dsLKR* for 48h were dehydrated at 80°C until a constant weight. Ten *H. cunea* larvae were weighed before and after dehydration using a Mettler MT5 analytical microbalance (Columbus, OH, United States). Water content was calculated as the difference between the fresh and dry weight. Each replicate contained 10 *H. cunea* larvae, and the experiment was performed in triplicate.

To study survival under desiccation and starvation, the *H. cunea* larvae treated with dsRNA were kept in empty vials or vials containing cotton ball with sterile water, respectively. Ten *H. cunea* larvae were used per replicate, and the experiment was performed in triplicate. The survival was recorded every 24h until all the *H. cunea* larvae were dead. The vials were placed in an incubator at 25±1°C under normal photoperiod conditions (16:8h light: dark).

### Food Intake Assay

On day of the seventh instar stage, *H. cunea* larvae were microinjected with dsRNA (*LK* and *LKR* dsRNA or *EGFP* dsRNA) and then returned to transparent plastic vials and starved for 24h. After a subsequent 4-day feeding period, the appetite of the larvae was checked by measuring the amount of artificial diet eaten by individual larvae during 24h. The weight of the artificial diet was measured before and after *H. cunea* larva feeding. Three biological replicates were included for each experiment, and for each biological replicate, 10 *H. cunea* larvae were kept in transparent plastic vials. The vials were placed in an incubator at 25°C under normal photoperiod conditions (16:8h light: dark).

### Quantitative Real-Time Reverse Transcription PCR

The RNA was extracted from *H. cunea* eggs, first to seventh instar larvae, pupae, adults, and tissue samples using the RNeasy Mini Kit (Qiagen, Valencia, CA, United States). The tissues – head, silk glands, midgut, epidermis, testis, ovary, Malpighian tubules, and fat body – were collected from larvae on day 1 of the seventh instar stage. cDNA was synthesized using the total RNA (0.5μg) and the PrimeScript®RT Reagent Kit with gDNA Eraser (Perfect Real Time, TaKaRa, Japan), according to the manufacturer’s protocol. The mRNA levels of *LK*, *LKR* and insulin-like peptide (*ILP*) genes were assessed using RT-qPCR with a SYBR Green kit (Toyobo, Osaka, Japan) and MJ Opticon™^2^ machine (Bio-Rad, Hercules, CA, United States). The reaction mixture (20μl) was composed of SYBR Green Real-time PCR Master Mix (10μl; Toyobo), nuclease-free water (7μl), gene-specific primers (1μl, 0.5μM; Table 1), and cDNA template (2μl; equivalent to 50ng of total RNA). *RPL13* and *EF-1α* were used as internal reference genes (Sun et al., 2019). The conditions for RT-qPCR reactions were as follows: 1cycle at 95°C for 30s, followed by 45cycles at 95°C for 12s, 60°C for 30s, 72°C for 40s, and 82°C for 1s for plate reading. The purity of the amplified products was analyzed by melting curve analysis. qRT-PCR was performed in using independent biological repeats in triplicate to ensure the reproducibility of the results. The expression levels of the clones were calculated using the 2^-ΔΔCt^ method (Livak and Schmittgen, 2001).

### Statistical Analysis

Statistical analysis was performed using SPSS (v17.0, SPSS Inc., Chicago, Illinois). One-way ANOVA was performed using Prism 8.0 (GraphPad Software, La Jolla, CA, United States). Value of *p*<0.05 was considered to indicate statistical significance for all experiments performed in the present study.

## Results

### HcLK and HcLKR Analyses

**Figure 1:**
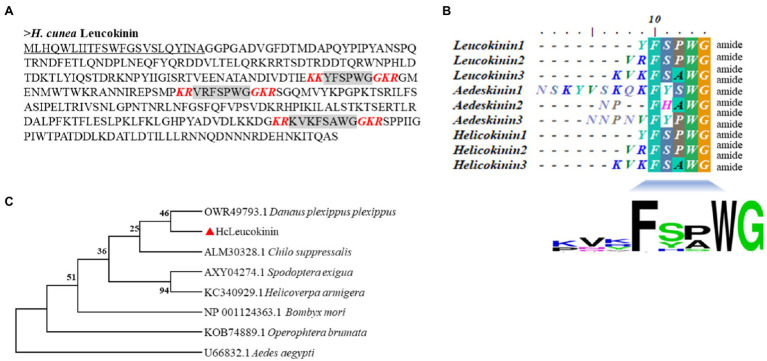
Comparison of the amino acid sequences of the *Hyphantria cunea* leucokinin (HcLK) precursor with those of other insect species. **(A)** shows the deduced amino acid sequence of HcLK. The underlined part of the sequence indicates the N-terminal putative signal peptide, and the gray part indicates the predicted HcLK peptide. The red bold and italic letters indicate the predicted amidation signals with dibasic cleavage sites. **(B)** shows an alignment of the consensus sequences of *H. cunea*, *Aedes aegypti*, and *Helicoverpa zea* putative, mature LK peptides. The calculated consensus logo is shown at the bottom. **(C)** shows cluster analysis of HcLK in various arthropods.

The sequences of *LK* and *LKR* genes were identified using transcriptome and genome analysis (Sun et al., 2019; Wu et al., 2019). The *LK* gene contains a 1,014bp open reading frame (ORF), which encodes a signal peptide (23 residues). The three mature peptide sequences comprise six (YFSPWGamide, HcLK-1), seven (VRFSPWGamide, HcLK-2), and eight (KVKFSAWGamide, HcLK-3) amino acid residues, respectively. The mature peptide cleavage site is a combination of lysine (K) and arginine (R) and has an amidation site “G” (Figure 1A). LK proteins from *H. cunea* and other insects showed very high sequence similarity (Figure 1B). Phylogenetic analysis revealed that HcLK and LKs from other insect species were clustered in a single group and that HcLK is most closely related to the *Danaus plexippus plexippus* homologs (Figure 1C).

**Figure 2:**
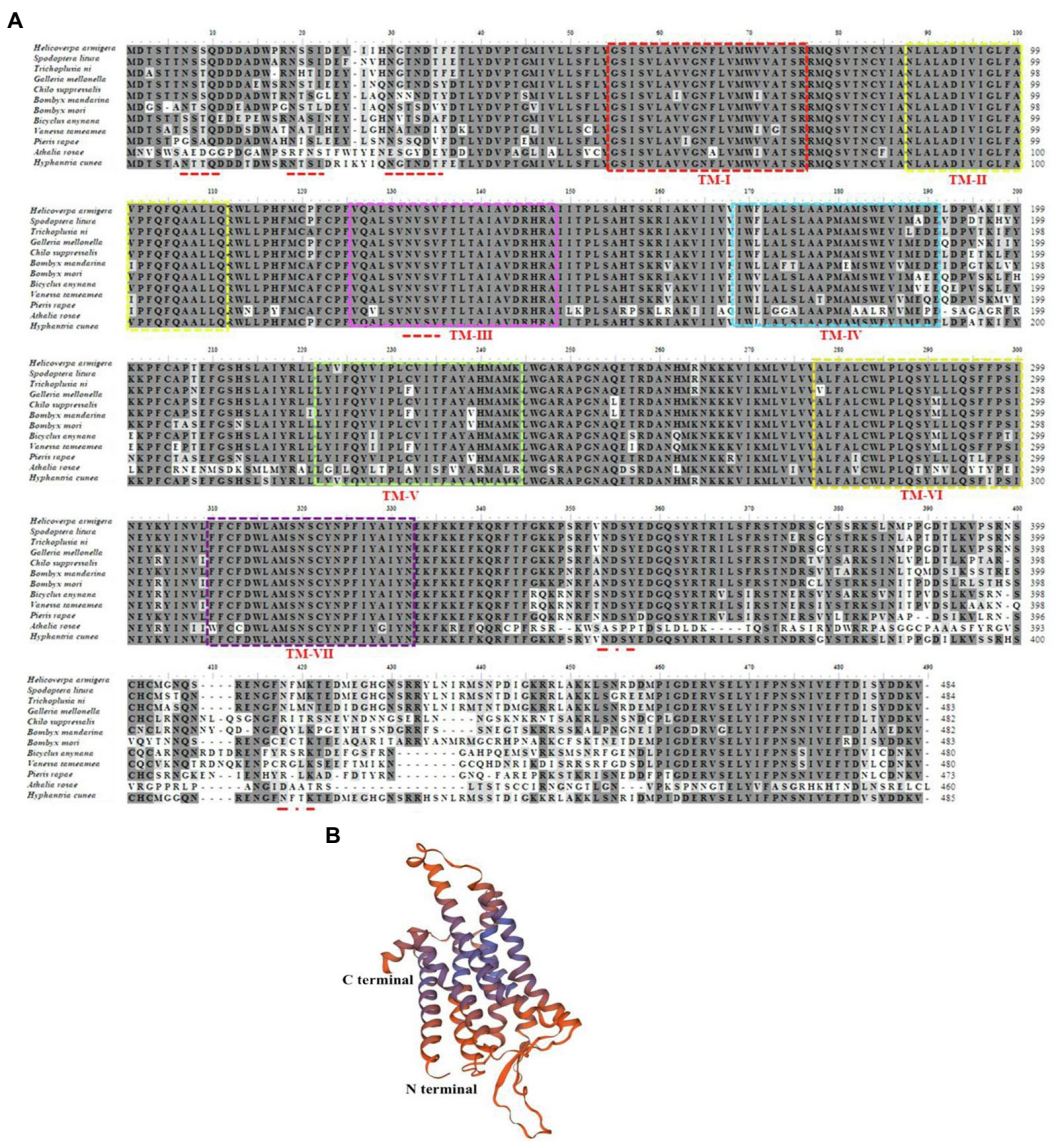
Amino acid sequence alignment of leucokinin receptor (LKR) in *Hyphantria cunea* with functionally deorphanized homologs in other insects **(A)** and predicted three-dimensional structure of mature HcLKR **(B)**. LKR sequences from the following species were used: *Helicoverpa armigera* XP_021201003.1, *Spodoptera litura* XP_022816525.1, *Trichoplusia ni* XP_026729237.1, *Galleria mellonella* XP_026751254.1, *Chilo suppressalis* ALM88319.1, *Bombyx mandarina* XP_028041362.1, *Bombyx mori* NP_001127721.1, *Bicyclus anynana* XP_023939936.1, *Vanessa tameamea* XP_026486140.1, *Pieris rapae* XP_022117160.1, and *Athalia rosae* XP_012267620.1. For ease of interpretation, identical residues are shaded black and conserved substitutions are shaded gray. The seven predicted transmembrane regions for all LKRs are marked with boxes of different colors. Putative N-glycosylation sites on the extracellular N–terminal domain of *H. cunea* LKR are indicated by red lines.

The full-length *HcLKR* cDNA consists of 2,186 nucleotides; the predicted ORF encodes 485 amino acids (Figure 2A). The ORF contains an ATG initiation codon, an upstream 608bp 5′ untranslated region (UTR), and a termination (TAA) codon followed by a 120bp 3′ UTR (Figure 2A). The HcLKR protein contains the characteristic seven transmembrane domains (TM, Figure 1B, TMHMM 2.0 server), with a typical signature of rhodopsin-like G protein-coupled receptor (GPCR; Figure 2A). The amino acid residues at positions 49–73, 82–104, 120–142, 163–179, 215–238, 266–291, and 306–331 represented TMI, TMII, TMIII, TMIV, TMV, TMVI, and TMVII, respectively. The predicted three-dimensional model of HcLKR showed a characteristic structure, with seven TM segments with α-helices (TM-I to TM-VII) linked by three intracellular and three extracellular loops, an extracellular amino terminus, and an intracellular carboxyl terminus (Figure 2B). Likewise, the Pfam analysis predicted seven transmembrane passes, and six conserved cysteine residues in the N-terminal extracellular domain. Six potential N-glycosylation sites were predicted for the N-terminal extracellular domain (NetNGlyc 1.0 server). Multiple amino acid sequence alignment between HcLKR and other LKRs showed high overall amino acid homology in the seven transmembrane domains (Figure 2B).

### Developmental and Tissue-Specific Expressions of *HcLK* and *HcLKR*

**Figure 3:**
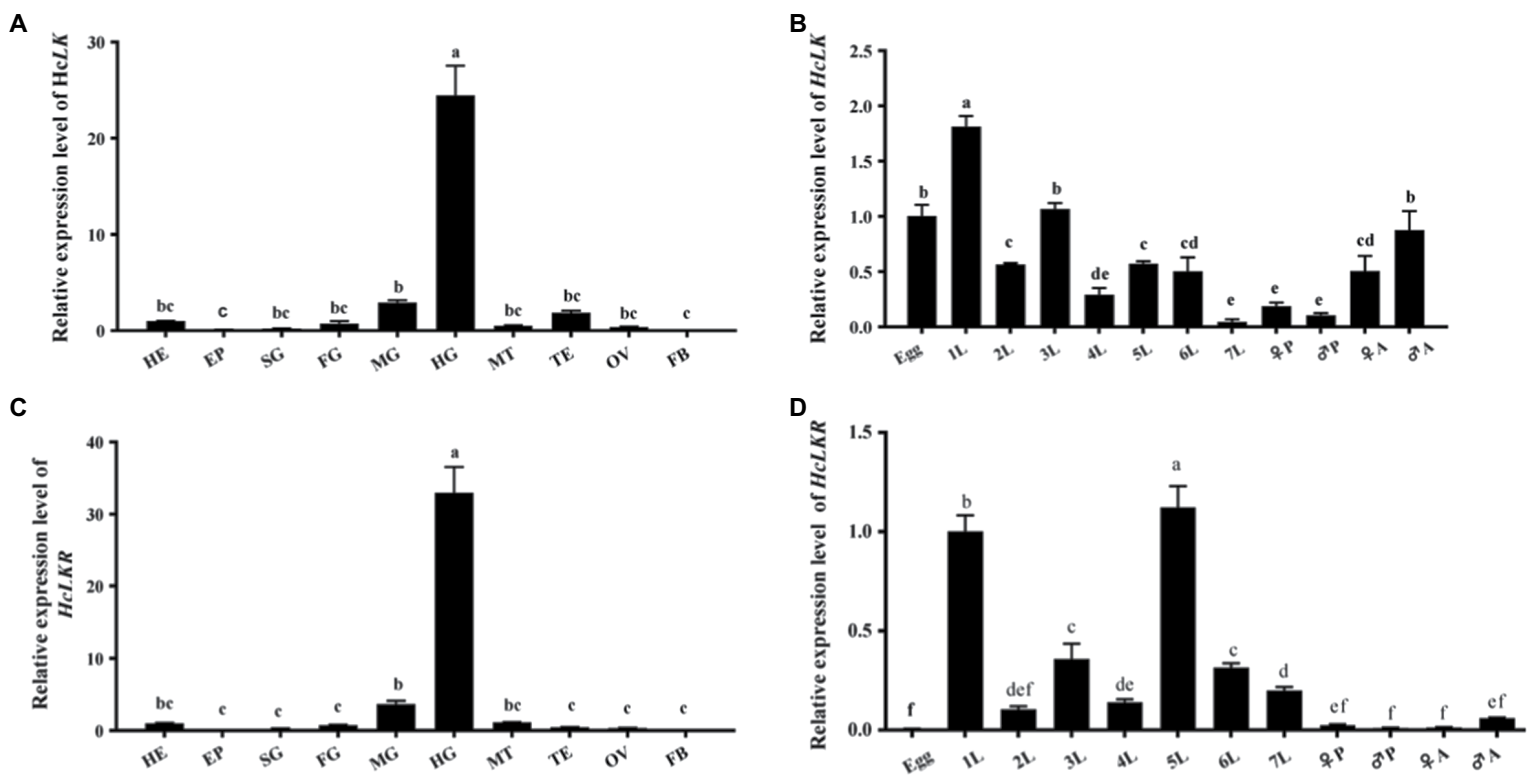
mRNA expression levels of *Hyphantria cunea* leucokinin and leucokinin receptor (*HcLK* and *HcLKR*) genes in *Hyphantria cunea*. **(A)** *HcLK* and **(C)** *HcLKR* mRNA expression levels in different tissues. Head (HE), epidermis (EP), silk gland (SG), foregut (FG), midgut (MG), hindgut (HG), Malpighian tubules (MT), testis (TE), ovary (OV), and fat body (FB) were dissected from *H. cunea* larvae on day 1 of the seventh instar stage; **(B)** *HcLK* and **(D)** *HcLKR* mRNA expression levels at various developmental stages. Each value is presented as mean±SD (*n*=3). Different lowercase letters (a–f) represent significant difference of *HcLK* and *HcLKR* levels among various tissues and developmental stages determined by one-way ANOVA followed by Tukey’s multiple comparison test (*p*<0.05).

The tissue-specific and developmental mRNA profiles of *HcLK* and *HcLKR* in *H. cunea* were quantified using RT-qPCR (Figure 3). Compared with that at the egg stage, the transcript level of *HcLK* in the first instar larvae was the highest (1.81-fold that in eggs) and that in the seventh instar larvae was the lowest (0.47-fold that in eggs). The expression level of *HcLK* in the hindgut was 24.45-fold of that in the head (Figures 3A,B). The expression of *HcLK* in silk gland, foregut, Malpighian tubules, testis, and ovary was 0.24-fold, 0.74-fold, 0.52-fold, 1.87-fold, and 0.38-fold of that in head tissue, respectively, and did not differ significantly. The *HcLKR* expression in the first and fifth larval stages was similar but significantly higher than that at other instar stages (*p*<0.05, Figure 3C). Compared with that in the head, the transcript level of *HcLKR* in the hindgut was the highest (32.96-fold that in the head and that in the fat body was the lowest 0.0004-fold that in the head). The *HcLKR* expression in the epidermis, silk gland, foregut, Malpighian tubules, ovary, and testis was 0.0004–1.16-fold that in the head (Figure 3D).

### Functional Activation of HcLKR

**Figure 4:**
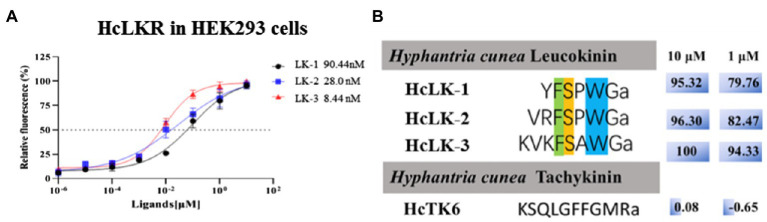
Ligand–receptor specificity of the *Hyphantria cunea* leucokinin receptor (HcLKR). **(A)** shows concentration-dependent response curves for HcLKR expressed in HEK293 cells induced by HcLKs. **(B)** shows ligand activity calculated on the basis of relative activity compared with the highest response of the receptor for HcLK-3.

The ORF of the *HcLKR* was inserted into the expression vector pcDNA3.1-Myc-His to construct a recombinant plasmid for stable expression. The *HcLK* gene encodes a 338-amino acid polypeptide (Figure 1A), which is a precursor of three LKs – LK-1–3 (Figure 4A). Notably, HEK293 cells expressing *HcLKR* responded to all HcLKs at a concentration of 1μM. The dose response of LKR to LKs was further investigated (Figure 4B). Of the three tested LKs, LK-2, and LK-3 stimulated LKR at lower concentrations, with EC_50_ values of 28.0 and 8.44nM, respectively, whereas LK-1 showed a lower activity (EC_50_ values: 90.44nM).

### Functions of HcLK and HcLKR by RNAi

**Figure 5:**
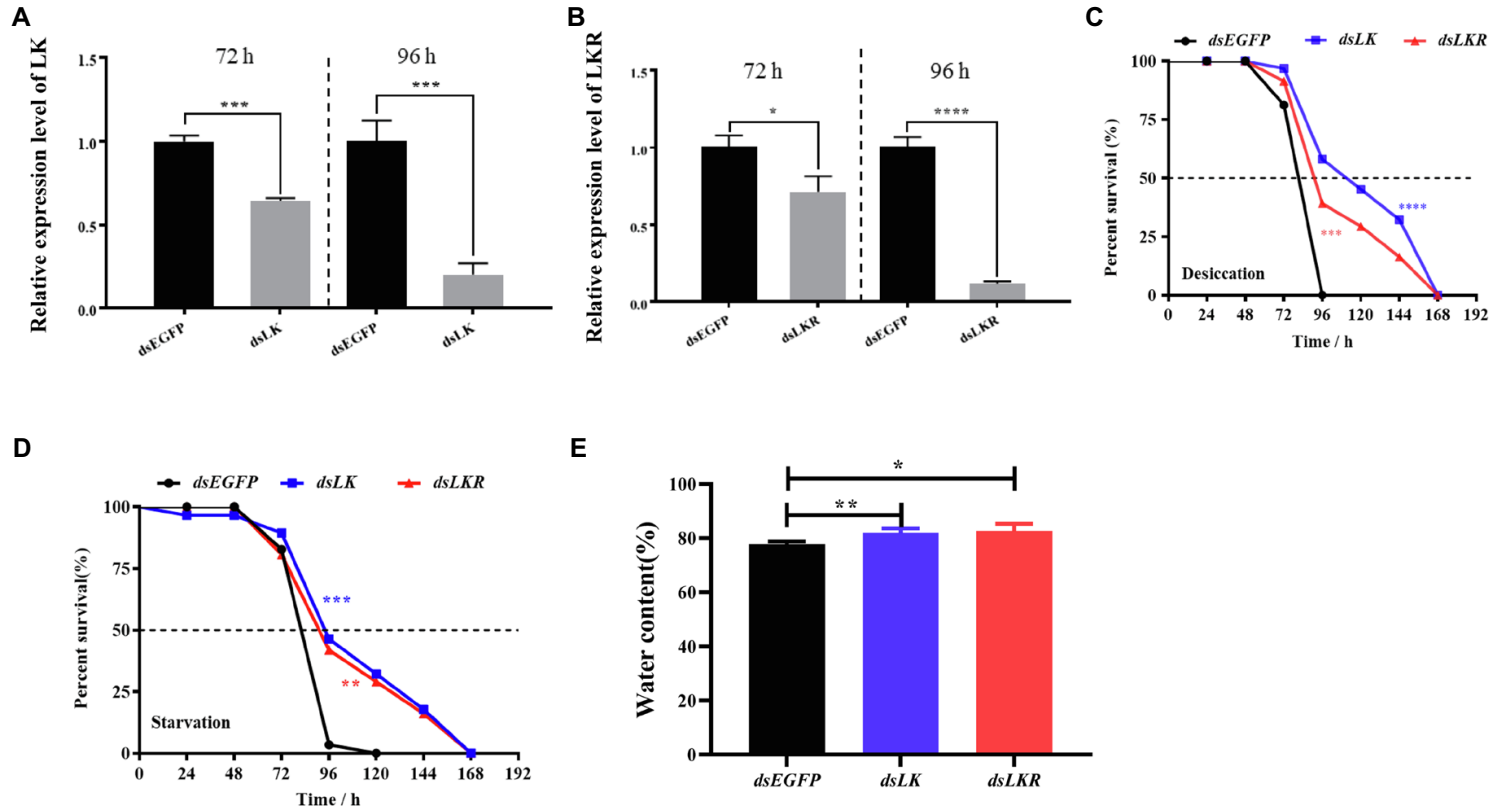
Effects of *LK* and *LKR* knockdown on seventh instar *Hyphantria cunea* larvae. **(A)** and **(B)** show effects of RNAi on gene expression of *LK* and *LKR* at 72 and 96h, respectively. Microinjection with double-stranded (ds) RNA targeting enhanced green fluorescent protein (EGFP) was used as a negative control. Values (mean±SD) are based on three biological replicates; each replicate contained pooled samples from four larvae. *p* values were calculated by unpaired *t*-test (^*^p<0.05, ^***^p<0.001, and ^****^p<0.0001). **(C)** and **(D)** show the survival rate of *H. cunea* larvae with *LK* or *LKR* knockdown under desiccation and starvation, respectively. Data are presented as survival curves, and the error bars represent SE [^**^p<0.01, ^***^p<0.001, and ^****^p<0.0001, as assessed by Log-rank (Mantel-Cox) test]. **(E)** shows water content of *H. cunea* larvae with *LK* and *LKR* knockdown. ^*^p<0.05 and ^**^p<0.01 as assessed by one-way ANOVA followed by Tukey’s multiple comparison test.

Considering the induction of *HcLK* and *HcLKR* mRNA expression by starvation stress, we investigated whether *HcLK* and *HcLKR* gene expression in the systemic silence plays a functional role in organismal stress tolerance employing knockdown of *HcLK* and *HcLKR via* dsRNA microinjection. The *HcLK* and *HcLKR* knockdown larvae showed ~80% lower *LK* and *LKR* mRNA levels than the control ds*EGFP* larvae after 96h (Figures 5A,B). Next, the survival of *H. cunea* RNAi larvae was investigated following desiccation and starvation stress.

Under desiccation and starvation stress, *HcLK* and *HcLKR* RNAi larvae survived longer than control larvae (Figures 5C,D). To determine whether the difference in survival rates of larvae stems from changes in water content, the water content in *H. cunea* larvae microinjected with ds*EGFP*, ds*LK*, and ds*LKR* were assayed after 48h of desiccation treatment. As expected, *H. cunea* larvae with ds*LK* and ds*LKR* silencing contained more water than those in control ds*EGFP* group (Figure 5E).

**Figure 6:**
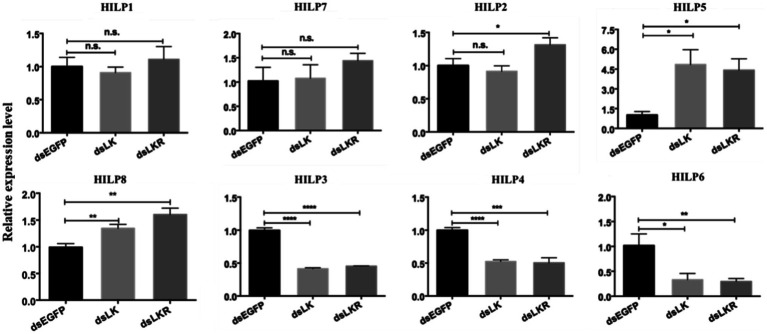
Effects of *HcLK* and *HcLKR* knockdown on insulin expression and starvation resistance. Values (mean±SD) are based on three biological replicates; each replicate contained pooled samples from four larvae. ^*^p<0.05, ^**^p<0.01, ^***^p<0.001, and ^****^p<0.001 as assessed by unpaired *t*-test. Values marked with “ns” are not significantly different (*p*>0.05; t test; n=3).

The expression of *ILP* genes in *H. cunea* was altered in ds*LK* and ds*LKR* larvae after 48h of starvation. Significant effects on *HILP* transcription were observed only for *HILP2* (except ds*LK* treatment), *HILP3*, *HILP4*, *HILP5*, *HILP6*, and *HILP8*. The transcript levels of *HILP2*, *HILP5*, and *HILP8* in the ds*LKR* larvae were significantly higher (1.31–4.42-fold) than those in the ds*EGFP* group. However, the transcript levels of *HILP3*, *HILP4*, and *HILP6* in the ds*LK* larvae were significantly lower (0.33–0.52-fold) than those in the ds*EGFP* group (Figure 6). Complex results were also observed when *LKR* and *LK* were knocked down in *H. cunea* larvae, the LK signal negatively regulated *HILP3*, *HILP4*, and *HILP6* expression but positively regulated *HILP5* and *HILP8* expression and played no significant regulatory role in *HILP1* and *HILP7* expression (Figure 6).

### *HcLK* and *HcLKR* Knockdown Promoting Feeding Behavior

**Figure 7:**
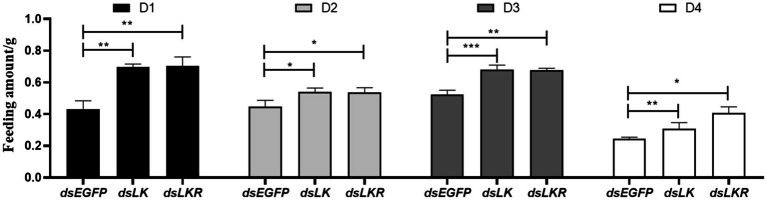
Effects of *LK* and *LKR* gene silencing on feeding behavior in *H. cunea*. Microinjection with ds RNA targeting *EGFP* was used as a negative control. Values (mean±SD) are based on three biological replicates. Each replicate contained pooled samples from 10 larvae. ^*^p<0.05, ^**^p<0.01, and ^***^p<0.001 as assessed by unpaired *t*-test (*n*=3).

Our results suggested that LK signaling is associated with starvation stress. Thus, the *HcLK* and *HcLKR* knockdown mutants were found to affect food intake over different periods. The food intake of larvae microinjected with ds*HcLK* and ds*HcLKR* after starvation for 1day was significantly different from that of larvae microinjected with dsEGFP (Figure 7). During the feeding time tested, the food intake of ds*HcLK* and ds*HcLKR* larvae was significantly higher than that of the control ds*EGFP* larvae. The food intake of ds*HcLK* and ds*HcLKR* larvae on the day 1 was 1.61- and 1.62-fold higher than that of the control ds*EGFP* larvae, respectively (Figure 7). On day 4 of feeding, the food intake of ds*HcLK* and ds*HcLKR* larvae was 1.26- and 1.66-fold higher than that of the control ds*EGFP* larvae, respectively.

## Discussion

Leucokinin, a multifunctional peptide acting as a neurohormone and neurotransmitter, is primarily synthesized in the CNS. Only a single *LK* gene was identified in *D. melanogaster* (Terhzaz et al., 1999). However, a single *LK* gene was first identified in *H. cunea*; which shares a similar typical structure of the LK family. Specifically, three putative LK proteins (LK-1–3) in *H. cunea* (HcLK-1–3) possess the general C-terminal motif sequence FxyWGamide (Veenstra et al., 1997). HcLK-1–3 showed high similarity with helicokinins 1–3 of *Helicoverpa zea*. The LKs are highly conserved between *H. cunea* and *H. zea* (Figure 1B). The *HcLK* genes were expressed in various tissues of *H. cunea*, especially highly expressed in the midgut and hindgut, as has been demonstrated in several insect species (Terhzaz et al., 1999; Kwon et al., 2016). In *Grapholita molesta*, *LK* was predominately expressed in the gut and FB (Cheng et al., 2021), whereas in *Chilo suppressalis*, *LK* were predominately expressed in the CNS and gut (Xu et al., 2016). Seven transmembrane domains involved in GPCR ligand binding and receptor activation are functionally conserved in *HcLKR*, which contains amino acid motifs typical of the GPCR family (Marco et al., 2013). Moreover, the isolated HcLKR was highly analogous to other LK receptors in various insect species. *HcLKR* was mostly expressed in the midgut and hindgut, as previously reported in *Aedes aegypti* and *D. melanogaster* (Kwon et al., 2016; Zandawala et al., 2018a,b). This phenomenon corresponds with the main function of LK in diuresis and ion transport (Gonzalez et al., 2012). The insect hindgut is the main organ of the excretory system. The highest expression levels of *HcLK* and *HcLKR* genes in the hindgut suggest a conserved function of the LK signaling system in the regulation of diuresis and ion transport (Coast et al., 2002; Dow and Davies, 2006; Nässel and Winther, 2010).

The intracellular Ca^2+^ levels were performed to determine the binding between HcLK peptides and HcLKR because Ca^2+^ acts as a second messenger for LKR signal transduction. Pharmacological data demonstrate that HcLKR was strongly activated by HcLK peptides in a concentration-dependent manner. Our results are consistent with the previously reported pharmacological characterization of LKR in *D. melanogaster* (Terhzaz et al., 1999; Radford et al., 2002).

The LK signaling system has been demonstrated to be involved in food intake, metabolism, and stress in insects (Al-Anzi et al., 2010; Liu et al., 2015; Zandawala et al., 2018a,b). Feeding or starvation affects the expression of *LK* and *LKR* in *D. melanogaster* (Zandawala et al., 2018a,b). Cannell et al. (2016) showed that, in *D. melanogaster*, starvation increases the epithelial *LKR* gene expression, and Malpighian tubule stellate cell-specific knockdown of *LKR* significantly reduces starvation tolerance. Zandawala et al. (2018b) showed that targeted knockdown of *LKR* in abdominal ganglion LK neurons using the CRISPR/Cas9 technology significantly increased starvation tolerance in in *D. melanogaster*. *LKR* mutation and targeted knockdown of *LKR* in insulin-producing cells of *Drosophila* altered the expression of ILPs and increased starvation resistance (Zandawala et al., 2018b). Yurgel et al. (2019) reported that the LK neuropeptide plays an essential role in the metabolic regulation of sleep. Moreover, the activity of LK neurons is modulated by feeding; decreased activity is observed in response to glucose, whereas increased activity is observed under starvation conditions. In the present study, our results showed that *LK* or *LKR* knockdown increased the water content in *H. cunea* and extended survival during desiccation and starvation. Under desiccation conditions, the survival rate of *H. cunea* larvae was improved by deletion of LK/LKR signaling, which promotes water retention. The findings confirm that the LK signaling system plays a vital role in the regulation of water homeostasis and the resistance to desiccation and starvation. The LK likely plays a regulatory role during starvation; however, its detailed functions remain to be identified. Moreover, *HcLK* and *HcLKR* knockdown increased the transcript levels of *HILP2* (except in the ds*LK* larvae), *HILP5*, and *HILP8* and decreased the transcript levels of *HILP3*, *HILP4*, and *HILP6*. However, *HcLK* and *HcLKR* knockdown had little effect on the transcript levels of *HILP1*, *HILP7*, and *HILP2* (except in the *dsLKR* treatment group). The LK/LKR system in *H. cunea* could be used to control *H. cunea* by synthesizing leucokinin analogs. However, the potential regulatory role of LK and LKR in the transcription of ILPs in *H. cunea* needs to be further studied.

## Data Availability Statement

The original contributions presented in the study are included in the article/Supplementary Material, further inquiries can be directed to the corresponding author.

## Author Contributions

LS and CC designed the research and wrote the manuscript. HM, YG, and ZW performed the experiments and analyzed the data. CC revised the manuscript. All authors contributed to the article and approved the submitted version.

## Funding

This work was funded by grants from the National Natural Science Foundation of China (32171791 and 31700570), the Fundamental Research Funds for the Central Universities (2572019CG04), the Natural Science Foundation of Heilongjiang (YQ2021C007), Heilongjiang Postdoctoral funds (LBH-Q20064), and Heilongjiang Touyan Innovation Team Program (Tree Genetics and Breeding Innovation Team).

## Conflict of Interest

The authors declare that the research was conducted in the absence of any commercial or financial relationships that could be construed as a potential conflict of interest.

## Publisher’s Note

All claims expressed in this article are solely those of the authors and do not necessarily represent those of their affiliated organizations, or those of the publisher, the editors and the reviewers. Any product that may be evaluated in this article, or claim that may be made by its manufacturer, is not guaranteed or endorsed by the publisher.

## Footnotes

^1^ http://www.cbs.dtu.dk/services/NetNGlyc/
